# Expiratory Muscle Strength Training for Drooling in Adults with Parkinson’s Disease

**DOI:** 10.1007/s00455-022-10408-6

**Published:** 2022-02-16

**Authors:** Naomi Cocks, Jonathan Rafols, Elizabeth Embley, Kylie Hill

**Affiliations:** 1 Curtin School of Allied Health, Curtin University, Perth, WA 6000 Australia; 2 ParkC Collaborative, Perth, WA Australia; 3 Royal Perth Bentley Group, East Metropolitan Health Service, Perth, WA Australia

**Keywords:** Parkinson’s disease, Drooling, Therapy

## Abstract

One of the most debilitating symptoms of advanced Parkinson’s disease is drooling. Currently, the main treatment that is offered for drooling is botulinum toxin injections to the saliva glands which have a number of side effects and do not treat the causes of drooling, such as impaired swallowing and lip closure. This study explored the effect of an alternative therapy approach for drooling that aimed at improving the swallow, expiratory muscle strength training (EMST). Sixteen participants received EMST over a 6- to 8-week period. Measurements were taken pre- and post-training for drooling (Sialorrhea Clinical Scale for Parkinson’s Disease; SCS-PD), swallowing, lip strength and peak cough flow. Measures of drooling, swallowing and peak cough flow were stable over pre-training assessments and improved following training (*p *< 0.01). The most conservative estimate of the within-group change for SCS-PD was − 2.50 (95% confidence interval −  3.22 to −  1.22). No adverse effects were reported and participants gave high satisfaction ratings for the training. A programme of EMST offers promise as a therapy to reduce drooling for people with Parkinson’s disease. Adequately powered randomised controlled trials of EMST are now needed.

## Introduction

Sialorrhea or drooling is a common consequence of Parkinson’s disease (Parkinson’s) with an estimated prevalence between 32 and 74% [1]. The cause of sialorrhea in Parkinson’s is not due to increased saliva production, but rather due to a combination of a number of factors including impaired swallow and decreased lip closure [2, 3].

The impact of sialorrhea on the lives of people with Parkinson’s is significant. Sialorrhea has been ranked as the third most bothersome symptom in advanced Parkinson’s [4] and is associated with poor health-related quality of life (HRQoL) and social isolation. Of particular concern, impaired swallow, which contributes to sialorrhea, has also been associated with impaired peak cough flow in people with Parkinson’s [5]. Taken together, sialorrhea and impaired swallow are likely to explain why this population is at increased risk of aspiration pneumonia, which is their leading cause of death [6].

Currently, sialorrhea is typically treated with botulinum toxin injections to the saliva glands which reduces the amount of saliva produced [7]. Although effective at reducing sialorrhea by reducing saliva production, there are a number of unpleasant side effects including exacerbating dysphagia, increased difficulties chewing and a dry mouth which in turn can result in degradation of the oral mucosa [8–11]. Given these side effects, research exploring alternative treatment options for sialorrhea is urgently needed. As sialorrhea in Parkinson’s is due, at least in part, to decreased impaired swallowing and lip closure, it follows that treatments that result in improved swallowing and improved lip strength may also result in reduced sialorrhea. One treatment that offers promise in this regard is expiratory muscle strength training (EMST). In EMST, the lips are placed around a hand-held device and then the person breathes out against a threshold load. To do this, they need to forcibly contract both their lip muscles to maintain a seal as well as their expiratory muscles to overcome the load. The main muscle responsible for lip closure is the orbicularis oris. It consists of both deep and superficial fibres [12]. Therapy that targets lip strength has been found to result in increased lip closure [13]. Furthermore, previous research has found that resistance training targeting the orbicularis oris has resulted in increased lip strength in participants who have had a stroke [14]. The action of maintaining lip seal during EMST provides a training load to orbicularis oris as the lips are required to form an airtight seal around the EMST device. Previous research has found increased activity of the orbicularis oris during EMST [15]. As such, this action should, in theory, serve to strengthen the muscles involved in lip closure and those involved in forced expiration.

Two studies have explored the effect of EMST in people with Parkinson’s [16, 17]. Troche et al. [17] carried out a randomised controlled trial (RCT) with 60 participants and Pitts et al. [16] conducted a single-group (no control group) study with ten participants. Both studies used the penetration and aspiration scale (PAS) and demonstrated significant improvements following EMST. In the RCT, the change in PAS was greater than any seen in the control group, reflecting an improvement in swallow function [17]. Additional benefits of following EMST included improvements in peak cough flow in the single-group study [16]. The effects on hyoid movement duration and maximum laryngeal closure assessed via videofluoroscopy were less clear [17]. Whilst these results provide preliminary data to suggest that EMST may improve swallow and peak cough flow, neither study explored the effects on sialorrhea and lip closure.

The current study explored the impact of EMST on a self-reported measure of sialorrhea. In order to explore the underlying mechanisms resulting in any improvement of sialorrhea, swallow function and lip strength were also measured. Changes in peak cough flow were also measured. It was hypothesised that there would be a reduction in sialorrhea, improved swallow function, increased lip strength and improved peak cough flow following EMST.

## Materials and Methods

This study obtained approval from the relevant Human Research Ethics Committees. The trial was registered with the Australian New Zealand Clinical Trials Registry (ANZCTR).

### Participants

Thirty-four adults who attended an Australian hospital with a diagnosis of Parkinson’s and who self-reported a difficulty with drooling were screened to participate in the research study. Seven declined to participate due to the time commitment required.

Participants were eligible to participate if they had a confirmed a diagnosis of Parkinson’s according to the UK Parkinson’s Disease Society Brain Bank Diagnostic Criteria [18].

Exclusion criteria were an inability to understand written or spoken English, evidence of cognitive impairment as indicated by a score of 17 or below on the Mini-Mental State Examination [19], botulinum toxin injections for drooling within the previous 8 months, and/or co-existing diagnosis that was likely to impact on sialorrhea or swallowing. Medical clearance was obtained from the participants’ doctors in order to rule out likely contra-indications for EMST. These included a recent history of broken ribs, pregnancy, untreated hypertension, untreated gastro-oesophageal reflux, severe asthma and acute stroke. Six participants were excluded.

Twenty-one participants consented to take part in the study. Five participants did not complete the study (24%). Two passed away, one became too unwell and two decided they no longer wished to participate. The results presented are of the remaining 16 participants.

The participants were predominantly male (1 female) consistent with a higher prevalence of Parkinson’s in the male population [20]. Participants ranged in age from 63 to 91 years. Time since diagnosis ranged from 2 to 15 years. The Levodopa Equivalent dose ranged from 188 to 1000 mg/day. Further demographic data are provided in Table 1.

**Table 1:** Participant characteristics

Participant #	Gender	Age (year)	Age of onset of Parkinson’s (year)	Levodopa equivalent daily dose (mg/day)
1	M	69	58	375
2	M	70	63	1000
3	M	74	68	250
4	M	66	57	188
5	M	85	79	800
7	F	53	45	1000
8	M	63	59	800
10	F	82	80	188
11	M	81	74	350
12	M	74	72	300
13	M	76	72	300
14	M	71	56	900
15	M	91	85	500
16	M	73	68	800
17	M	77	66	800
20	M	84	78	500
21	M	90	87	300

### Protocol

This study comprised three components. The first component (A) involved four pre-training assessment sessions which were completed over 1 to 2 weeks. The second component (B) constituted the intervention period, during which participants completed 30 sessions of EMST over 6 to 8 weeks. Each week, training comprised two supervised sessions (face to face with a speech pathologist) and three sessions at home (unsupervised). The third component (C) involved two post-training assessment sessions which were completed over the first week immediately following completion of component B.

### Measures

The primary experimental measure, the Sialorrhea Clinical Scale for Parkinson’s Disease (SCS-PD) [21], was used to measure the severity of sialorrhea. During component A, to obtain a double baseline for this measure, the SCS-PD was completed twice, 1 week apart. It was collected once following training. This questionnaire has been found to be valid and reliable [21]. Higher scores on the SCS-PD represent worse drooling.

The Mann Assessment of Swallowing Ability (MASA) [22] was used to assess severity of swallowing function and the Swallowing Quality of Life Questionnaire (SWAL-QOL) [23] was used to assess health-related quality of life (HRQoL) associated with swallowing. During component A, to obtain a double baseline for this measure, the MASA was completed twice, 1 week apart. The SWAL-QOL was assessed once prior to training. The MASA and SWAL-QOL were collected once following training. These assessments are reliable, valid and responsive [23–26]. Lower scores on the MASA and the SWAL-QOL represent greater difficulty with swallowing and impact on HRQoL.

Lip strength measures were collected using the Iowa oral performance instrument (IOPI Medical LLC, Washington, USA). Peak expiratory flow was measured during a cough manoeuvre using a hand-held flow meter (EasyOne spirometer, NDD Medical Technologies, Massachusetts, USA). During component A, measures of lip strength and peak cough flow were collected during four sessions, each separated by ≥ 24 h. During each assessment session, participants were asked to perform 10 efforts for each measure. The best measure that was within 10% of at least two others was recorded as the result for that assessment session. Lip strength and peak cough flow were collected once following training.

On study completion, participants were asked to rate the level of satisfaction with the training. The rating was based on a 10-point scale ranging from completely unsatisfied (0) to completely satisfied (10). Adverse effects reported by participants were also recorded.

### Intervention

During each session, EMST was conducted using a threshold loading device (EMST 150 device, Aspire Respiratory Products, North Carolina, USA). Prescription of the initial load was based on the principles of resistance training for adults described in the position stand by the American College of Sports Medicine [27]. Specifically, it is recommended that older adults (and otherwise sedentary people) commence resistance exercise at loads equivalent to 40 to 50% of maximum. Hence for the initial training session, we aimed to have participants reach a load equivalent to 50% of the peak pressure that could be generated (by their expiratory muscles). This approach has been shown to be feasible in an earlier randomised control trial of inspiratory muscle training in people with chronic obstructive pulmonary disease [28]. Thereafter, training loads were titrated according to symptoms. Although it is possible that higher training loads could have been tolerated and produced greater gains, this may have compromised their satisfaction with the training programme.

Participants performed the EMST in a seated position. Only one participant required a nose clip for the first two face-to-face therapy sessions to prevent leakage of air through the nose. All participants were instructed to breathe at their usual rate and depth. An interval-based training approach was used to optimise the training load that could be tolerated [28]. Specifically, participants were asked to breathe with a single forced expiration, followed by a 15-s rest. This was repeated 25 times with a one-minute rest every five breaths. At the end of each five breath work interval, participants were asked to rate their perceived exertion, using the 0 to 10 Borg category ratio scale [29]. Training loads were progressed as quickly as possible with the goal of having the load during the final two-minute work interval perceived as ‘very hard’ (7/10 on the Borg scale) with participants unable to consistently maintain lip closure during their last few expiratory efforts.

### Analyses

For measures that were collected multiple times during component A, one-way repeated measures analysis of variances (ANOVAs) were used as has been used in similarly designed research studies (for example see [30]). If the assumption of sphericity was violated, the Huynh–Feldt Epsilon adjustment was applied. For outcomes that were assessed once during component A, paired t-tests were used to compare the pre- and post-training measures.

## Results

### Implementation Fidelity

All 16 participants attended all face-to-face therapy sessions. Fifteen participants adhered to the training protocol for all face-to-face sessions. One participant developed an illness during the intervention period and missed four sessions. He had his training period extended by 2 weeks and these sessions were rescheduled. No adverse events were noted throughout the study.

All participants who completed the study reported being satisfied with the training programme. Twelve participants gave the maximum rating of 10 (very satisfied). The remaining four participants gave a rating of 9.

### Outcome Measures

The mean differences between the pre- and post-training measures and 95% confidence intervals (CI) for these mean differences are presented in Table 2.

**Table 2:** Mean differences and 95% confidence intervals for differences

Measure and time points	Mean difference	95% confidence interval
Sialorrhea Clinical Scale for Parkinson’s disease		
Pre-training 1 vs post-training	− 2.50*	− 3.22 to − 1.22
Pre-training 2 vs post-training	− 3.06*	− 4.37 to − 1.75
Mann assessment of swallowing ability		
Pre-training 1 vs post-training	4.50*	0.87 to 8.13
Pre-training 2 vs post-training	4.62*	1.90 to 7.35
Swallowing quality of life questionnaire (SWAL-QOL)		
Pre-training vs post-training	7.64*	3.37 to 11.92
Lip strength (kPa)		
Pre-training 1 vs post-training	3.62	− 0.55 to 7.8
Pre-training 2 vs post-training	4.00*	1.14 to 6.86
Pre-training 3 vs post-training	5.06*	1.05 to 9.07
Pre-training 4 vs post-training	3.87	-0.22 to 7.96
Peak cough flow (L/min)		
Pre-training 1 vs post-training	55.09*	28.93 to 81.25
Pre-training 2 vs post-training	46.28*	26.4 to 66.15
Pre-training 3 vs post-training	37.31*	17.07 to 57.55
Pre-training 4 vs post-training	34.57*	14.37 to 54.77

*Delineates significant difference at *p *< 0.01

### Sialorrhea

When all pre- and post-training SCS-PD scores were included in the analysis, there was an effect for time, *F*(2,30) = 24.39, *p *< *0.01, partial* *η*^2^ = *0.62.* Pairwise comparisons revealed that there were no differences between the two pre-training scores (*M* = 10.31, SD = 3.11; *M* = 10.87, SD = 3.22) and the post-training score (*M* = 7.81, SD = 3.29) was significantly less than both pre-training scores. This indicated that self-reported saliva difficulties significantly decreased post-training.

### Swallow

When all pre- and post-training MASA scores were included in the analysis, there was an effect for time, F(1.56, 23.59) = 7.58, *p *< *0.01,* *η*^2^ = *0.336.* Pairwise comparisons revealed that there were no difference between the two pre-training scores (*M* = 182.56, SD = 9.86, *M* = 182.44, SD = 8.96) and the post-training score (*M* = *187.06, *SD = 7.91) was significantly higher than both pre-training scores, indicating an improvement in swallow function.

For SWAL-QOL, compared with the pre-training scores (*M* = 72.04, SD = 11.59), the post-training scores (*M* = 79.68, SD = 9.69) were significantly higher, *t*(15) = − 3.8, *p *< *0.01*).

### Lip Strength

When all pre- and post-training measures of lip strength were included in the analysis, there was an effect for time, *F*(4, 60) = 6.82, *p *< *0.01,* *η*^2^ = *0.312.* Pairwise comparisons revealed that there were significant differences between the post-training measures (*M* = 28.25*, *SD = 7.12) and the measures collected during the second and third pre-training sessions (*M* = 24.25, SD = 6.26; *M* = 23.19, SD = 6.27) but not the measures collected during the first and fourth pre-training sessions (*M* = 24.62, SD = 5.72; *M* = 24.37, SD = 6.48).

### Peak Cough Flow

When all pre- and post-training measures were included in the analysis, there was an effect for time, *F*(4, 60) = 9.45, *p *< 0.01, *η*^2^ = *0.386*. Pairwise comparisons revealed that there were no differences between the four measures collected pre-training (*M* = 4.18, SD = 1.82; *M* = 4.33, SD = 1.95; *M* = 4.48, SD = 1.99; *M* = 4.52, SD = 2.15) and the post-training measure (*M* = 5.104, SD = 2.14) was significantly higher than each pre-training measure.

## Discussion

In people with Parkinson’s, the results of this single-group study demonstrated that the measure of sialorrhea was stable over the pre-training assessment period but improved following a period of EMST. These data are encouraging and provide a strong foundation to justify a future RCT to demonstrate the effect of EMST on sialorrhea by optimising swallowing and lip closure. That is, this study provides preliminary data to support an alternative therapy approach to botulinum toxin injections for sialorrhea with no reported side effects.

All participants indicated that they were highly satisfied with EMST as a treatment for sialorrhea. Attendance at face-to-face treatment was high and there was an improvement in HRQoL associated with swallowing. This suggests that the treatment approach was well tolerated by participants and was not considered burdensome.

Furthermore, unlike botulinum toxin injections which do not treat the cause of sialorrhea, EMST was found to result in improvements to some of the underlying factors that contribute to sialorrhea, namely reduced swallow efficiency and reduced lip closure. The results of this research extend the results of previous studies on EMST in Parkinson’s [16, 17] by showing that this training produced within-group differences in swallow function, thus targeting the pathophysiological mechanism underlying drooling.

Our data also suggest that there is an important additional benefit of using EMST for sialorrhea treatment, improvement in peak cough flow. Aspiration pneumonia is the most common cause of death for people with Parkinson’s [6] and the development of aspiration pneumonia risk is associated with swallowing impairment and impaired cough [13–15]. The impact of EMST on aspiration pneumonia risk is yet to be systematically explored. However, as EMST targets physiology responsible for both swallow function and peak cough flow, and aspiration pneumonia is usually as a result of aspiration of contents due to an impaired swallow and a reduced ability to expectorate aspirate, it is likely that EMST will result in a reduction in aspiration pneumonia risk. This should be explored in future research.

Whilst this study makes an important contribution to the current body of research on sialorrhea, there were a number of limitations. The main sialorrhea measure used was a self-reported measure. Whilst there is currently no research which suggests that awareness of sialorrhea is impacted, reduced awareness of motor symptoms has been found to be a difficulty for many people with Parkinson’s [31]. As such, a self-reported measure may not be the most accurate measure for some people with Parkinson’s. However, it is important to note that validity of the SCS-PD was demonstrated by comparing to saliva volume measures [21] and currently, there are no other published measures of sialorrhea suitable for people with Parkinson’s with superior psychometric properties [32]. Similarly, this study used the MASA [21] which is a bedside assessment of swallow and is dependent on the clinician’s observations. Future research should include a videofluoroscopic swallowing study as a more objective measure of swallow function. However, it is important to note that many aspects of the interpretation of videofluoroscopic swallowing studies are also dependent on the clinician’s observations and judgement. The sample size was modest and the 95% confidence intervals around our estimate of the differences for some outcomes, such as peak cough flow, are quite wide. To definitively examine the effect of EMST on these outcomes, an adequately powered randomised controlled trial (RCT) is needed that incorporates blinding of participants (by offering sham training to a control group). Attrition was consistent with other behavioural treatments for such a debilitating condition. The rate of attrition should be taken into consideration when carrying out sample size calculations for the RCT.

In conclusion, this study served as an important first step in exploring the impact of EMST on sialorrhea for people with Parkinson’s. The results indicate that EMST results in a reduction of sialorrhea and that this is likely due to improvements in swallow function. This study provides an important first step to identifying an alternative treatment to botulinum toxin injections for sialorrhea with no side effects, high satisfaction and improved HRQoL.
